# Enhancing laboratory biosafety management: a comprehensive strategy from theory to practice

**DOI:** 10.3389/fpubh.2024.1439051

**Published:** 2024-09-20

**Authors:** Qin Tang, Fei Yan, Lu Yuan, Ying Tang, Hui Chen, YuTing Sun, Mi Yang, GuoLin Song

**Affiliations:** ^1^Department of Clinical Laboratory, The Fourth People's Hospital of Chengdu, Chengdu, China; ^2^MOE Key Lab for Neuroinformation, Department of Clinical Laboratory, The Clinical Hospital of Chengdu Brain Science Institute, University of Electronic Science and Technology of China, Chengdu, China

**Keywords:** laboratory biosafety, quality system documentation, risk assessment, infection control system, evaluation system, BSL-2

## Abstract

**Objective:**

This study examines biosafety management practices in a psychiatric hospital’s laboratory in China, focusing on how outdated information technology impacts the hospital’s ability to respond to public health emergencies. The goal is to enhance the hospital’s emergency response capabilities by updating risk assessments, biosafety manuals, and implementing a comprehensive quality management system alongside a specialized infection control system for significant respiratory diseases.

**Methods:**

We utilized an integrated research approach, expanding the scope of risk assessments, updating the biosafety manual according to the latest international standards, and implementing a quality management system. A specialized infection control system for significant respiratory diseases was introduced to improve emergency response capabilities.

**Results:**

Updated risk assessments and a new biosafety manual have significantly improved the identification and management of biosafety threats. Implementing new quality management and infection control systems has enhanced response efficiency and operational standardization.

**Conclusion:**

The measures taken have strengthened the biosafety management and emergency response capabilities of the laboratory department, highlighting the importance of information technology in biosafety management and recommending similar strategies for other institutions.

## 1 Introduction

Amid the increasing frequency of global public health events and the rapid advancement of biotechnology, the significance of laboratory biosafety management has become increasingly prominent. Particularly in China, with the swift progress of biotechnology and the growing number of public health emergencies, biosafety management has risen to the level of national strategy (1). In 2020, the Standing Committee of the National People’s Congress passed the “Biosafety Law of the People’s Republic of China,” marking the first time that China has systematically regulated biosafety management through legislation. This law encompasses several domains, including the biosafety of pathogen laboratories, genetic technology safety management, and the prevention and control of invasive species, laying the legal foundation for biosafety management in China. In addition, China addresses the management of bioagents through the “Regulations on the Biosafety Management of Pathogenic Microorganism Laboratories” (issued in 2004, State Council Order No. 424) and the “Regulations on the Biosafety Management of High-Pathogenic Pathogen Laboratories” (issued in 2004, State Council Order No. 424, revised in 2005). These regulations provide clear standards for the handling of different types of pathogenic microorganisms within various biosafety laboratories (e.g., BSL-2, BSL-3, BSL-4) and outline the requirements for laboratory facility management. Furthermore, the National Health Commission issued the “General Requirements for Laboratory Biosafety” (GB19489-2008), and the Chinese Center for Disease Control and Prevention (CDC) has formulated multiple biosafety technical guidelines and operational specifications, such as the “Technical Specifications for Biosafety Laboratory Construction” (GB50346-2011) and the “Technical Specifications for the Transportation of High-Pathogenic Pathogenic Microorganisms” (WS233-2002). These laws and standards provide comprehensive guidance for laboratory operations, ensuring strict compliance with biosafety protocols. In China, BSL-2 laboratories in specialized and grassroots hospitals play a crucial role in detecting, identifying, and preventing potential biological threats. However, these laboratories still face numerous challenges in biosafety management, which not only hinder the advancement of biosafety standards but also limit their ability to enhance emergency response capabilities (2).

1. *The lag in information technology*: Biosafety management in BSL-2 laboratories plays a crucial role in safeguarding public health and research security. However, many clinical laboratories in specialized and primary care hospitals face a prominent challenge—insufficient integration of information systems (3). This issue is particularly evident in the lack of integrated information systems between laboratories, infection control departments, and clinical departments, resulting in significant management gaps that compromise overall biosafety levels. Specifically, laboratories rely on traditional methods, such as telephone notifications and manual recording, to handle reports on infectious diseases and multidrug-resistant organisms. This approach is not only inefficient but also prone to errors. Moreover, many laboratories are unable to accurately record specimen transit times (TAT) through information systems, making it extremely difficult to monitor key pre-analytical quality indicators in real-time. This lag in information systems not only leads to delays in data updates but also makes real-time monitoring and early warning for infectious diseases and multidrug-resistant organisms unfeasible. The reliance on manual data processing means that laboratories face highly complex tasks in handling statistics related to infectious diseases, multidrug-resistant organisms, abnormal susceptibility results, and non-compliant specimens, which heavily depends on manual collection and analysis. This situation not only increases the risk of reporting delays and data errors but also severely hampers the laboratory’s ability to identify and respond to potential biosafety threats in a timely manner. Additionally, the lack of interoperability in information systems further hinders effective collaboration with infection control departments and clinical departments, limiting the comprehensive understanding and sharing of biosafety data. This fragmented state not only exacerbates data silos but also impedes the full implementation of comprehensive prevention and control measures, rendering the laboratory more powerless and inefficient when facing complex biosafety challenges.
2. *Insufficient risk assessment:* Despite national standards requiring risk assessments, the understanding of these assessments among laboratory personnel is often inadequate, leading to an imperfect risk assessment process, especially in identifying high-risk pathogens, thereby increasing the risk of biosafety incidents (4). The lack of a standardized risk assessment system means that potential biological threats are often overlooked during the identification of high-risk pathogens, and potential hazards in specimen handling and laboratory operations are not fully understood.
3. *The emergency system is imperfect:* The current manual recording and reporting processes are prone to errors, making it difficult to handle biosafety incidents effectively and timely. Without comprehensive emergency plans, laboratory personnel are unable to undergo adequate training and drills, preventing them from responding swiftly to biological threats (5). Although emergency documentation has been developed, most remain in the preparatory phase, and laboratory personnel often lack a deep understanding or have misconceptions, leading to non-compliance with procedures, further reducing the efficiency of biosafety emergency handling.
4. *The management system is not comprehensive:* The existing biosafety management system lacks cohesion and dynamism, and the inadequate mechanisms for interdepartmental collaboration lead to difficulties in sharing biosafety data. The laboratory is unable to fully grasp the current state of biosafety risks or adjust management strategies in a timely manner. Due to the fragmented structure of the management system, the lack of information flow and resource coordination between departments makes it difficult for the laboratory to respond quickly to incidents, weakening the overall level of protection and affecting cooperation with other departments (6).

To address these challenges, this study aims to update the biosafety manual and develop a comprehensive quality management system with a special emphasis on advancing information technology, improving risk assessment processes, and optimizing emergency systems. By establishing interconnected information systems and refining the process for identifying high-risk pathogens, this study expects to significantly enhance the laboratory’s level of biosafety management and emergency response capabilities, providing effective prevention and control strategies against future biological threats.

## 2 Research background

In China’s specialized and primary hospitals, the importance of laboratory biosafety management is becoming increasingly evident, particularly against the backdrop of frequent global public health events and ongoing laboratory-related infection incidents (7). Recent studies indicate that laboratory personnel are at risk of exposure to infectious pathogens that can cause conditions ranging from asymptomatic infections to life-threatening diseases. For instance, Lin et al. (8) emphasize the necessity of stringent biosafety emergency management measures when researching emerging infectious diseases like SARS-CoV-2 to ensure the safety of laboratory personnel and the environment. Alderman et al. (9) highlight the critical need for biosafety plans in animal experimentation, as laboratory workers are at risk of exposure through direct contact with experimental animals or infected microbes. Additionally, Qasmi and Khan (10) found that common sources of laboratory infections in Karachi include needlestick injuries, animal bites, and mucosal cuts, all of which pose significant risks of laboratory-associated infections. A global “risk-driven” strategy underscores the importance of basing improvements in laboratory biosafety on biosafety manuals, adjusting technical specifications, and standardizing personal protective equipment and behavioral protocols to enhance laboratory biosafety (11). In the level 2 biosafety laboratory of the hospital where the researchers are based, pathogens such as *Pseudomonas aeruginosa*, *Staphylococcus aureus*, and *Acinetobacter baumannii*, common nosocomial pathogens, have been detected on the hands of staff members, and methicillin-resistant *Staphylococcus aureus* (MRSA) has been found on microbiology lab surfaces. These findings reveal potential biosafety hazards in the laboratory, further highlighting deficiencies in the current laboratory biosafety management system in terms of risk assessment, emergency preparedness, and operational protocols.

Despite the existence of numerous national and international standards and guidelines for laboratory biosafety management, laboratories continue to face significant challenges in their implementation. For example, Mohsen and Dpagh (12) noted that 80% of laboratory infection incidents could be attributed to human errors, highlighting the importance of rigorous biosafety risk assessments. Peng et al. (13) further emphasized the necessity of adopting evidence-based biosafety measures and strategies to address associated infection risks. During the early stages of the COVID-19 pandemic, deficiencies in the laboratory personnel training systems significantly impacted the emergency response capabilities of laboratories, particularly due to the lack of routine emergency drills, which led to uncertainty and panic among laboratory personnel when handling emerging pathogens. A study found that during the early stages of the pandemic, the absence of professional training and practical guidance on the novel coronavirus resulted in considerable anxiety and concern among laboratory staff when processing viral samples, directly affecting their work efficiency and compliance with safety operations (14). In Wuhan, China, where the COVID-19 virus outbreak initially exploded, many laboratory workers, having never encountered such a virus before, lacked targeted training and emergency drills. Some local laboratories experienced hesitation and non-standard practices in the early stages of the outbreak due to inadequate risk assessments and emergency response training, increasing the risk of cross-contamination. This situation somewhat delayed the processing speed of samples and the efficiency of virus detection, affecting the overall effectiveness of epidemic control (15). Additionally, the lack of routine emergency drills meant that laboratory personnel were not sufficiently proficient or correct in using personal protective equipment (PPE), leading to unnecessary exposure risks. Reports indicated that in some laboratories, individual staff members caused self-contamination incidents by not strictly adhering to the standard procedures for donning and doffing protective gear (16). These examples underscore the importance of enhancing regular training and emergency drills for laboratory personnel during public health crises. Regular drills simulating real epidemic response scenarios can not only improve laboratory personnel’s awareness and adherence to biosafety measures but also boost their confidence and ability to handle emerging pathogens. Therefore, it is recommended that laboratories strengthen targeted education and training to ensure all staff can respond quickly and accurately in emergency situations, effectively reducing biosafety risks.

Based on existing research, laboratory biosafety management in specialized and primary hospitals still faces the following challenges:

1. *Technological backwardness*: Many hospital clinical laboratories lack integrated information systems with infection control departments and clinical departments, relying on telephone notifications and manual registration for reporting infectious diseases and multidrug-resistant organisms, resulting in low efficiency and accuracy. Additionally, the turnaround time (TAT) for specimen transport cannot be accurately tracked through information systems, and critical quality indicators before testing are difficult to ascertain. Moreover, data collection and statistical analysis rely on manual processes, making it difficult to automate information statistics and infectious disease alerts, thereby weakening the efficiency and effectiveness of biosafety management.
2. *Limitations of biosafety manuals*: The content of current biosafety manuals is overly general and not updated in a timely manner, particularly lacking in specific Standard Operating Procedures (SOPs), which leaves laboratory personnel without reliable operational guidance. For example, there is a lack of detailed protective guidance for handling high-risk pathogens, making it difficult for laboratories to flexibly respond to evolving biological threats. Moreover, the manuals lack a timely update mechanism, failing to cover emerging risk factors, which diminishes their practicality in real-world applications.
3. *Deficient training systems*: Many laboratory personnel lack targeted biosafety training, struggling to grasp the latest risk assessment and control knowledge and skills. The absence of a regular emergency drill system reduces the confidence and accuracy of laboratory staff when facing crisis events. The lack of a continuous training system prevents staff from adequately mastering the correct use of Personal Protective Equipment (PPE) and from performing protective operations according to standardized procedures.
4. *Insufficient integration and dynamism in management systems*: The existing management systems lack real-time monitoring and evaluation mechanisms, making it difficult for laboratories to comprehensively grasp current biosafety risk conditions and adjust management strategies promptly. Additionally, the lack of integration in the management systems, inadequate inter-departmental collaboration, and insufficient information-sharing mechanisms hinder communication and resource sharing with infection control departments and clinical units, weakening overall biosafety protection and rapid response capabilities.
5. *Inadequate capacity to handle high-risk pathogens*: The existing biosafety management systems in specialized and primary care hospitals face numerous challenges when dealing with high-risk pathogens that require BSL-3 or BSL-4 laboratory testing (17). Firstly, the lack of information system support hinders hospitals from achieving real-time sample tracking and coordination with higher-level laboratories, thus compromising the efficiency and safety of specimen transport processes. Secondly, laboratory personnel often lack proper training aligned with BSL-3 and BSL-4 standards, and the improper use of protective equipment further increases potential biosafety risks. Moreover, the absence of well-established mechanisms for interdepartmental collaboration and information sharing impedes resource integration and the ability to respond swiftly to high-risk pathogens. This, in turn, weakens the hospital’s responsiveness and protective measures in the event of biosafety incidents.

Based on these challenges, this study aims to update the biosafety manual and construct a comprehensive quality management system, enhancing the application of information technology to improve the level of biosafety management in secondary biosafety laboratories in primary or specialized hospitals. By establishing interconnected information systems, this approach facilitates real-time reporting and sharing of information on infectious diseases and multi-drug-resistant organisms. Developing a comprehensive monitoring system ensures the transparency and traceability of pre-test quality indicators. Additionally, by utilizing advanced data analysis techniques to optimize the identification and risk assessment processes for high-risk pathogens, this study seeks to significantly enhance the capability of hospital laboratories to handle biosafety risks and improve their response to public health emergencies.

## 3 Research methods and data collection

This study used a diversified methodological approach to ensure a comprehensive assessment and improvement of the laboratory biosafety management system. The first step was to expand the scope of existing risk assessments (11) to include a broader range of biosafety factors. Simultaneously, the current biosafety manual was updated to align with the latest international biosafety management standards. Additionally, a comprehensive quality management system was implemented to standardize laboratory operational procedures.

Data collection in this study was conducted through three primary methods: standardized assessments, systematic observations, and a specialized infectious disease control system for psychiatric healthcare institutions. Firstly, a standardized assessment process was implemented to accurately measure the effectiveness of laboratory biosafety measures. This process included regular self-assessments and peer reviews to quantitatively analyze compliance with biosafety procedures, incident handling effectiveness, and preventive measures. These assessment tools were meticulously designed to objectively reflect the true level of laboratory biosafety management (18). Secondly, continuous monitoring of daily laboratory operations facilitated data collection on the actual application of biosafety measures. This included staff compliance with biosafety protocols, correct use of protective equipment, and the efficiency of emergency response activations. Observational results helped identify potential risk points and non-compliant behaviors, providing a basis for further risk mitigation strategies (8). Lastly, the introduced hospital infection control system offered an advanced data monitoring platform that not only automatically recorded events related to biosafety but also enabled timely adjustments and optimizations of biosafety management measures through its real-time feedback and effect tracking capabilities. This comprehensive method of data collection ensured a holistic evaluation of the new biosafety measures from various perspectives, thereby enhancing the laboratory’s overall response capability and management efficiency to biological threats. Data analysis was conducted using quantitative methods to evaluate the effectiveness of newly introduced biosafety measures and adjust various risk indicators and standards based on real-time data analysis. Ultimately, all collected data were used to generate a comprehensive risk assessment report and periodically evaluate the effectiveness of the implemented biosafety measures. These methods not only enhanced the laboratory’s emergency response capabilities to public health emergencies but also improved the overall efficiency and effectiveness of biosafety management (19).

## 4 Improvement measures

### 4.1 Transformation and enhancement of the quality management system

#### 4.1.1 Requirement analysis and benchmark setting

During the requirement analysis and benchmark setting phase, a comprehensive assessment of the current laboratory biosafety management system is conducted to identify key areas for improvement. Ensuring that the management system meets evolving international standards and best practices is crucial. International standards such as ISO 15189: 2012 and ISO 9001:2015 specify requirements for the structure, facilities, equipment, and data analysis of clinical laboratories, and provide guidelines for the structure of quality manuals, division of responsibilities, and documentation of operational procedures. Additionally, CWA 15793 offers guidance on risk assessment, control measures, and continuous improvement for bio-risk management. To establish a management system that aligns with international standards, an initial review of past biosafety records and internal assessment reports is performed to identify weaknesses and ensure consistency in internal and external reviews and bio-risk assessments. Learning from the experiences of other leading international laboratories and institutions also informs the benchmark setting process (20). Based on this foundation, the objectives for the improved system are set, including specific goals in risk identification, data reporting, personnel training, and standardization of procedures. Key performance indicators (KPIs), such as compliance, error rates, and sample processing times, are defined. Through real-time monitoring and periodic reviews, the new management system is ensured to remain aligned with international standards and undergo continuous improvement.

#### 4.1.2 Structural restructuring and framework development

During the structural restructuring and framework development phase, the quality management system is divided into four hierarchical levels to ensure that each level adequately covers essential aspects of biosafety management. Initially, at the strategy level, it is necessary to formulate and clarify the overall policies, objectives, and management framework for laboratory biosafety, providing clear guiding principles and overall direction to ensure that the overall strategy meets the needs of the laboratory. Following this, at the procedural level, comprehensive biosafety procedures are established, covering risk assessment, emergency response, and training development, thereby establishing standardized processes and operational norms to guide actual laboratory operations. Subsequently, at the operational level, detailed operational guidelines and Standard Operating Procedures (SOPs) are developed to guide laboratory personnel through each step of daily safety practices, minimizing potential safety risks within processes. Finally, at the records and reporting level, a robust system for documenting and reporting is necessary to effectively track biosafety events and the implementation of improvement measures, ensuring that each improvement is scientifically and promptly reflected in the management system (21). Through this four-tiered document structure, the quality management system integrates comprehensiveness and attention to detail, achieving comprehensive standardization of laboratory biosafety management.

#### 4.1.3 Content creation and quality review

Led by the laboratory department, this phase involves collaboration among the medical department, infection control department, infectious disease wards, microbiology professionals, and pharmacy staff to ensure that the management system addresses the practical needs of all relevant specialties. Initially, different departments work together to draft detailed content for each level of documentation, ensuring that Standard Operating Procedures (SOPs) and biosafety policies accurately reflect best practices across various professional fields. The team also integrates international standards with current practices to refine the documents down to specific operational steps, ensuring the accuracy and practicability of the guidelines. In terms of quality review, an interdisciplinary team examines the completeness and logic of each level of documentation, with particular attention to the applicability of SOPs in laboratory processes, to ensure seamless integration into practical operations.

#### 4.1.4 Personnel training and strategy deployment

**Figure 1:**
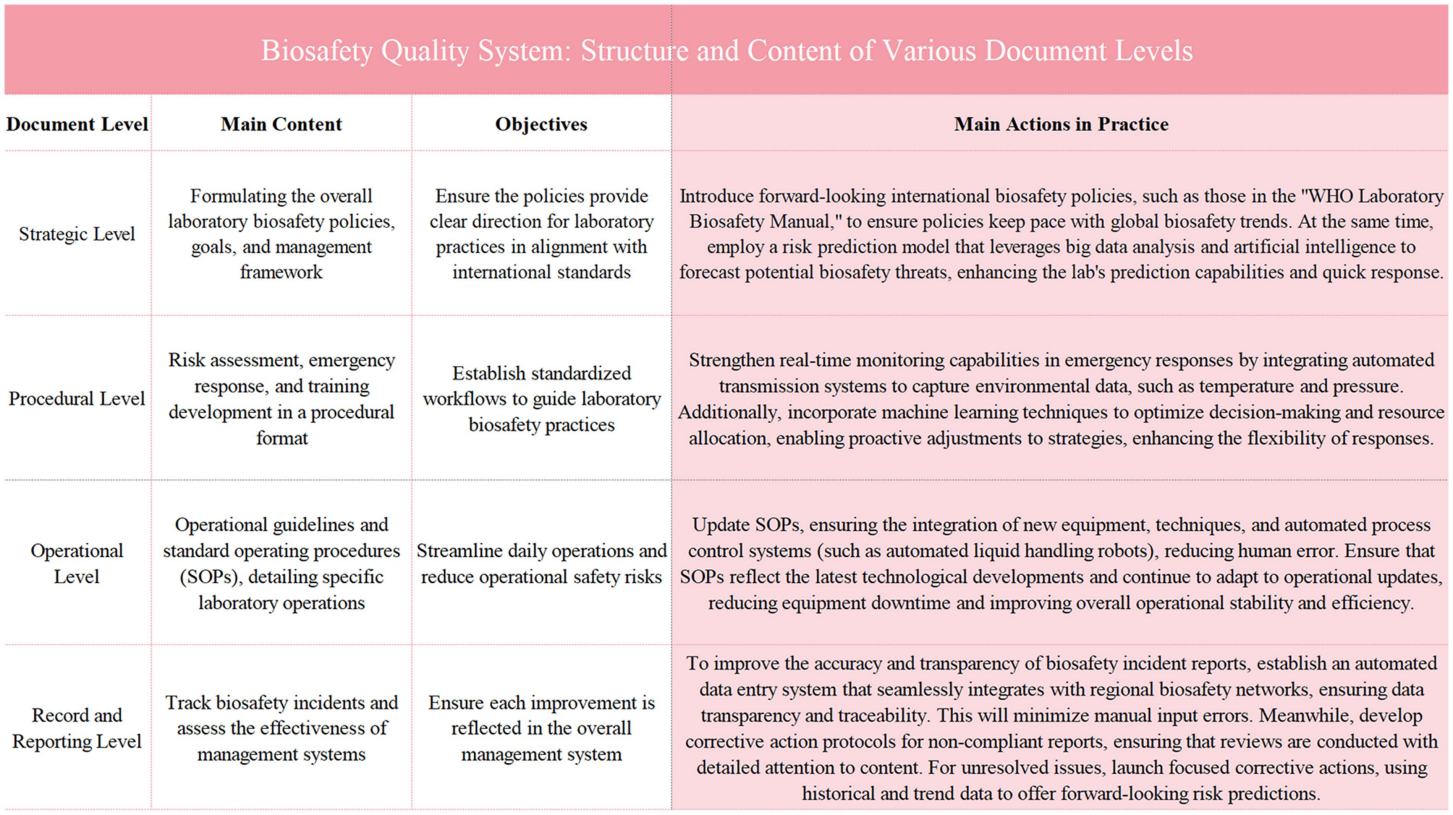
Illustrates the structure and content of various document levels within the biosafety quality system. It outlines the main content, objectives, and practical actions taken at the strategic, procedural, operational, and record and reporting levels. Each level contributes to the overall enhancement of biosafety management by ensuring policy alignment with international standards, establishing standardized workflows, updating operational procedures, and improving the tracking and reporting of biosafety incidents. The figure emphasizes the integration of advanced technologies and systematic frameworks to optimize laboratory safety and response capabilities.

Organizing comprehensive training for all relevant personnel is crucial to ensure that every member fully understands the new system and can apply it to laboratory processes. The training is organized by the team that developed the quality management system and combines lectures, demonstrations, and hands-on practice to ensure that everyone accurately masters the new standards and procedures. Training content includes various levels of the new quality management system and specific Standard Operating Procedures (SOPs), guiding staff to accurately implement them in daily laboratory practices. Additionally, the training emphasizes critical aspects such as risk assessment, sample handling, emergency response, and data reporting, to ensure standardized operations during these processes. Detailed explanations and drills cover the key points, risks, and countermeasures of each process, ensuring the accuracy and safety of operations. Regarding strategy deployment, training is integrated with actual laboratory workflows to develop feasible implementation plans. Initially, key areas requiring adjustment in current processes are identified to ensure that the new management system integrates seamlessly with existing operations. Subsequently, an implementation schedule is established, deploying the new system in phases to ensure smooth operation during the transition period. Thirdly, by fostering teamwork, a monitoring and feedback mechanism is set up to promptly collect issues and suggestions for improvement during implementation, continuously optimizing the execution of the new quality management system (22). Ultimately, personnel training and strategy deployment ensure that the new quality management system is effectively implemented within laboratory processes, enhancing the standardization and sustainability of biosafety management (Figure 1).

### 4.2 Comprehensive update of the risk assessment system

**Figure 2:**
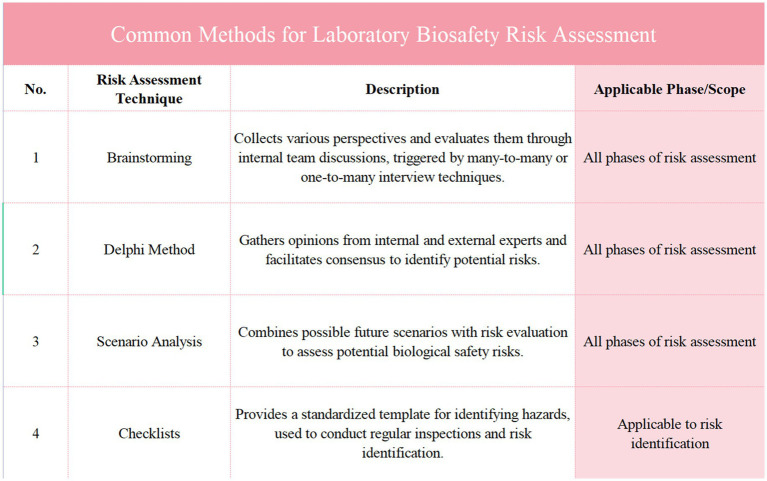
Presents an analysis of major risk assessment standards and their applications. It compares key documents, including the WHO laboratory biosafety manual, ISO 9001:2015, and national standards from China, outlining their date of issue, issuing body, main content, and impact or application. This figure highlights the global alignment of biosafety risk management guidelines and their implementation across various sectors, providing a comprehensive overview of the frameworks that guide laboratory biosafety practices, risk assessments, and management strategies in different industries.

In recent years, with the increasing frequency of global public health events, risk identification and assessment have become indispensable components of laboratory management. The introduction of various risk assessment standards reflects not only the international community’s emphasis on laboratory biosafety but also demonstrates that standardized risk assessment processes can significantly enhance a laboratory’s ability to respond to emerging pathogens and other biosafety threats (23). Both WHO’s global guidelines and China’s domestic standards provide comprehensive risk assessment frameworks, assisting laboratories in optimizing their management from risk identification to risk response (Figure 2).

The design of the risk assessment system update is intended to ensure that biosafety management measures can promptly respond to changes in the laboratory environment and potential threats (32, 33). This process is divided into two key phases: broad classification and standard setting, and dynamic assessment and reporting mechanisms.

#### 4.2.1 Broad classification and assessment standard setting

Initially, a comprehensive classification of potential biosafety risks faced by the laboratory is conducted, including categorization based on types of biological hazards (such as bacteria, viruses, fungi, and parasites) and risk factors (such as operational complexity, exposure probability, and severity of consequences). Each risk category is graded according to its potential threat to laboratory safety, such as high, medium, or low risk. Specific weights are assigned to each risk level during the standard setting, based on the potential consequences and probability of occurrence of the risks (24). For instance, management and control measures for high-risk pathogens are assigned higher weights to reflect their significant threat to laboratory safety. The setting of weights is based on past accident data, literature reviews, and expert experience, ensuring the scientific validity and practicality of the assessment system.

#### 4.2.2 Dynamic assessment mechanism and report generation

**Figure 3:**
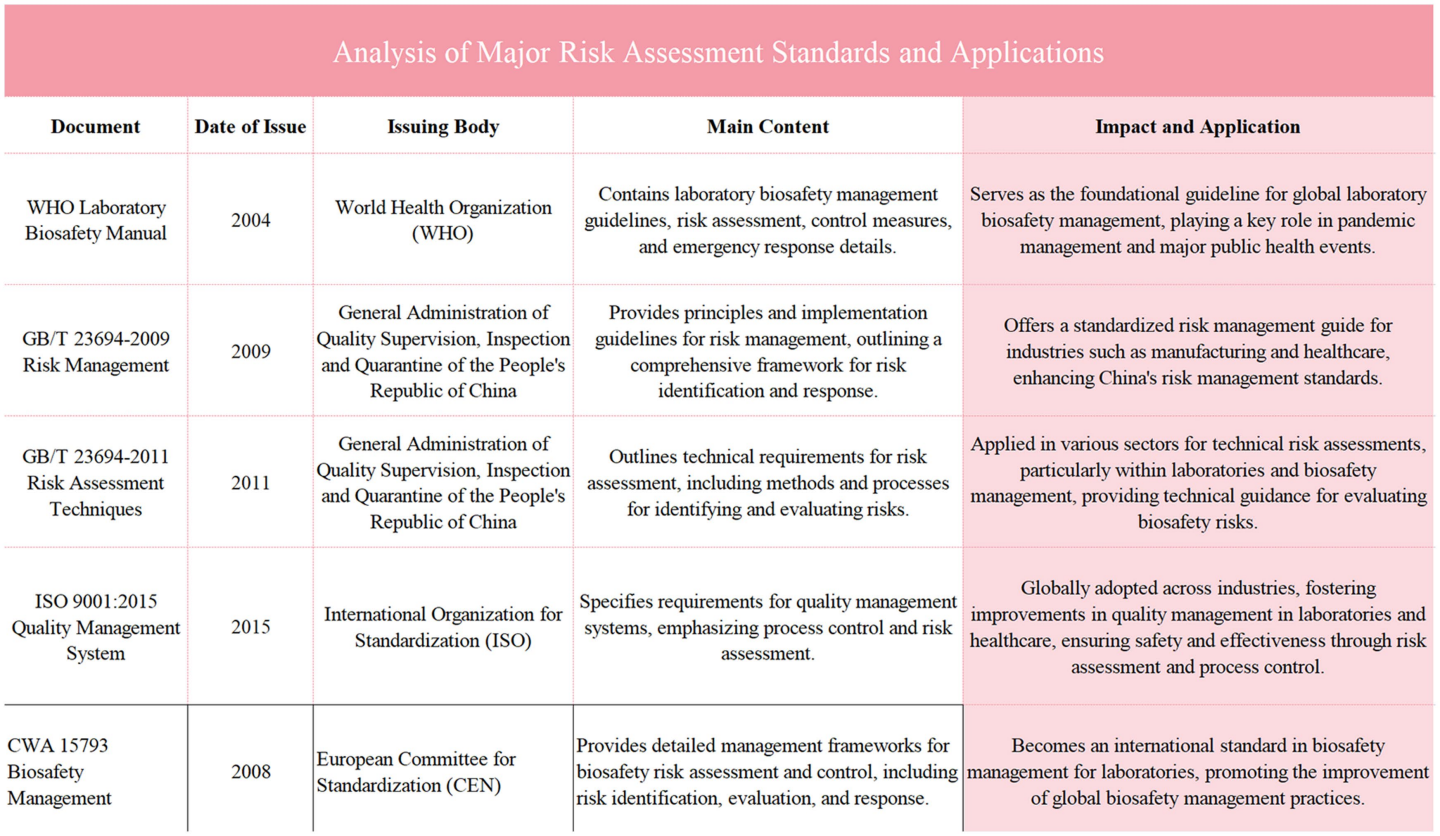
Illustrates the common methods for laboratory biosafety risk assessment, including techniques such as Brainstorming, the Delphi Method, Scenario Analysis, and Checklists. Each method is described based on its purpose in identifying and evaluating biosafety risks across various phases of risk assessment, ranging from general brainstorming and expert consensus methods to scenario-based evaluations and standardized checklists for hazard identification. This table provides an overview of how these methods are applied in laboratory settings to enhance risk management and ensure comprehensive safety measures are in place.

The risk assessment system employs a dynamic updating mechanism, adjusting the risk assessment standards through real-time data and ongoing monitoring. Dynamic adjustments are made by weighted calculations of various risk indicators, taking into account new research findings, technological advancements, or internal changes in the laboratory, such as the introduction of new equipment or process adjustments. For example, if the mode of transmission of a pathogen becomes more complex, the risk weight associated with that pathogen will be increased. Data collected during the assessment process includes laboratory incident reports, regular safety audit results, and data related to international biosafety events. These data are input into a central database and processed using specialized analysis software to generate a risk assessment report. The report not only indicates the current level of risk but also recommends necessary preventive measures and improvement strategies (25). Such an assessment system ensures the continuity and adaptability of laboratory biosafety management, able to promptly reflect any internal or external changes affecting biosafety, thus ensuring the safety of laboratory personnel and the integrity and effectiveness of experiments (Figure 3).

### 4.3 Innovation and application of the major respiratory infectious disease hospital infection control system

#### 4.3.1 System design and module integration

**Figure 4:**
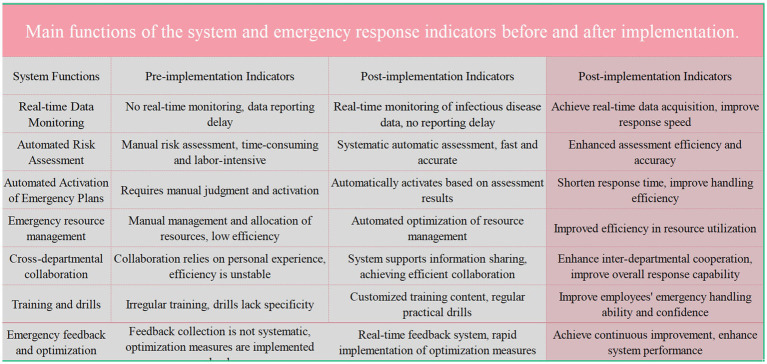
Compares the key functions of the system and emergency response indicators before and after the implementation of a new system. The pre-implementation indicators highlight issues such as the lack of real-time data monitoring, manual risk assessments, and inefficient emergency resource management. Post-implementation indicators show significant improvements including real-time data monitoring, automated risk assessments, and enhanced inter-departmental collaboration, resulting in improved response speed, efficiency, and resource utilization.

To address the unique characteristics and significant respiratory infectious disease control needs of specialty health care institutions, the system adopts a data-driven design philosophy, integrating modules such as simulation drills, real-time feedback, and performance tracking to optimize decision-making and operational efficiency (Figure 4). The simulation drill module replicates various scenarios of respiratory disease outbreaks, including patient admission, isolation ward management, specimen collection, and emergency medical procedures. Additionally, specialized scenario-based training for high-risk pathogens was introduced, covering simulations of BSL-3 and BSL-4 laboratory standard operating procedures. This training helps staff master complex skills such as the proper use of personal protective equipment (PPE), handling high-risk pathogen samples, and emergency management. During simulations, staff must execute actions according to drill protocols to gain authentic experience in responding to sudden public health events. The module also sets up multiple levels of outbreak scenarios, from localized outbreaks to large-scale ones, enabling staff to master response techniques at all levels (26). During the simulation drills, the real-time feedback module monitors the operation process, generating instant operational records and error reports, providing targeted guidance to each employee to help them correct mistakes and optimize procedures. Monitoring data is transmitted synchronously to the management backend, offering a comprehensive view during drills to identify and improve any weaknesses in the process. Additionally, the system includes a performance tracking module, This module conducts a comprehensive analysis of staff operational records accumulated during simulation exercises, identifying their strengths and weaknesses in infectious disease prevention and control. This analysis provides data support for subsequent training and the optimization of operational procedures. Additionally, the training content is customized based on employees’ years of service. For those with <5 years of experience, the focus is on foundational operational skills and the correct use of personal protective equipment (PPE). For employees with over 5 years of experience, training emphasizes emergency decision-making, management processes, and handling complex infectious disease outbreak scenarios. This personalized training approach, based on years of service, ensures that each employee receives targeted training appropriate to their experience level, effectively enhancing their response capabilities.

#### 4.3.2 System implementation process

**Figure 5:**
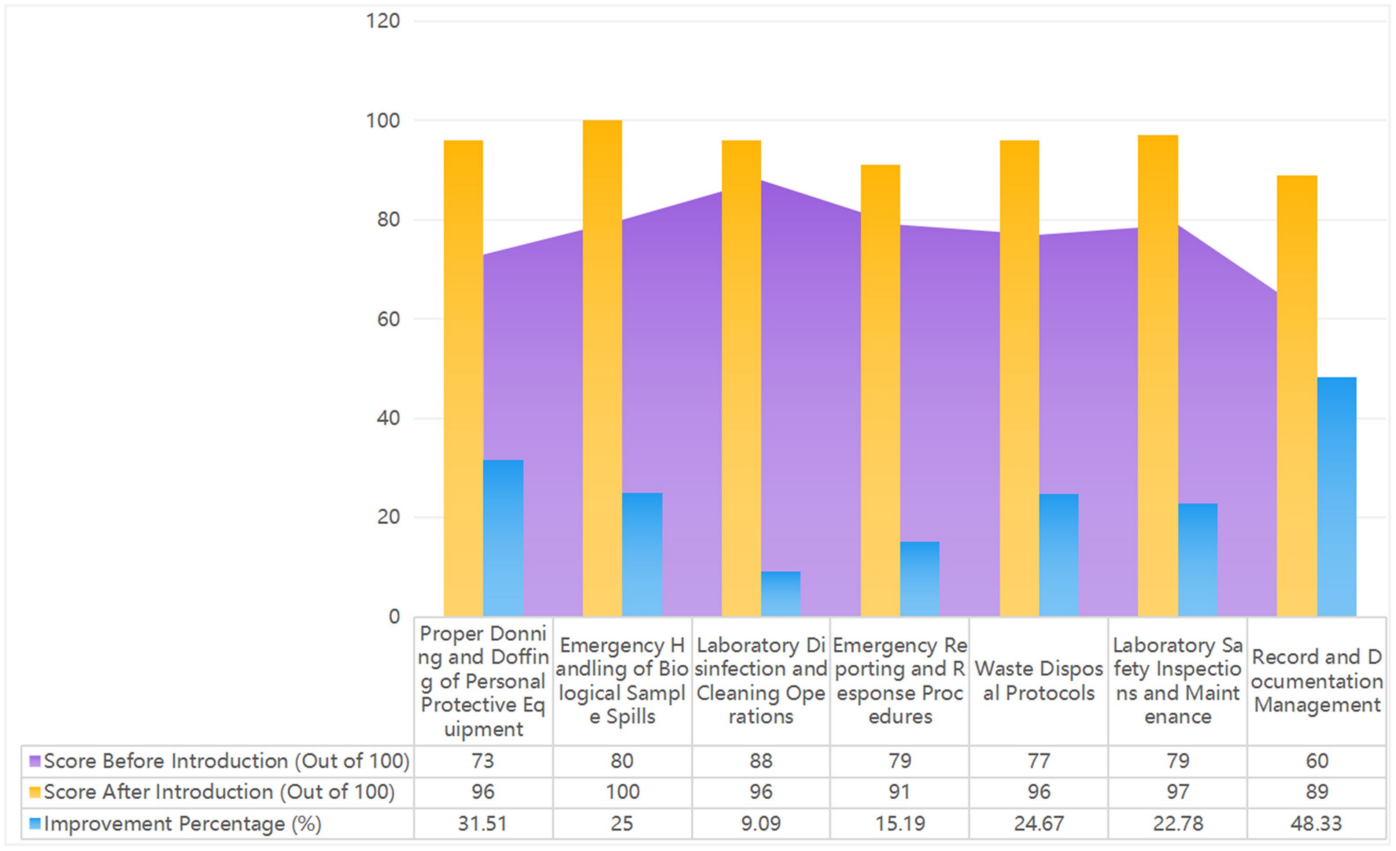
Shows the 2023 laboratory biosafety evaluation results, with scores out of 100. It compares system functions and emergency response indicators before and after implementing the Major Respiratory Infectious Disease Hospital Infection Control System. The figure highlights improvements in real-time data monitoring, automated risk assessments, and emergency resource management, demonstrating the enhanced efficiency and effectiveness of the new system.

Catering specifically to the characteristics of mental health care institutions, we have introduced the “Major Respiratory Infectious Disease Hospital Infection Control System for Mental Health Institutions, “aimed at enhancing the hospital’s capability to control and respond swiftly to significant respiratory diseases using technological means. The system’s design incorporates data-driven decision support and operational optimization to ensure efficient management during sudden public health events. It consists of three core modules: the Simulation Drill Module, Real-Time Feedback Module, and Performance Tracking Module. The Simulation Drill Module creates multi-level infectious disease outbreak scenarios ranging from small-scale local outbreaks to large-scale transmissions, allowing staff to learn and respond to complex situations in a controlled environment (27). This system is designed to enhance the practical operational skills and emergency response speed of the staff while simulating various challenges they might encounter in real scenarios. The Real-Time Feedback Module monitors the performance of staff during simulations, providing immediate operational suggestions and corrective actions, helping them swiftly enhance their skills in action. This module provides management with immediate feedback on employee performance and process effectiveness through detailed data analysis, enabling more precise management decisions. The Performance Tracking Module evaluates the entire team’s performance and progress through comprehensive analysis of drill data, identifying strengths and weaknesses in disease control. These analyses provide a scientific basis for future training and operational optimization, ensuring the hospital can continuously enhance its level of biosecurity management. The system’s implementation process starts with a needs analysis to ensure each step aligns with the hospital’s actual requirements. The installation and configuration stage requires close cooperation across multiple departments to adjust and optimize the system for the specific medical environment. After the installation and configuration of the system are completed, a comprehensive training program will be initiated to ensure that all relevant personnel can proficiently operate the system and understand its application in real working environments. The training for laboratory staff is customized based on their years of experience. For newly hired staff, the focus will be on basic operational skills, biosafety protocols, and the correct use of personal protective equipment (PPE). More experienced staff, with longer tenure, will receive in-depth training on emergency management, responding to complex infectious disease outbreaks, and system decision-making processes. The training content will include both online learning and assessments, as well as offline course instruction, ensuring a close integration of theoretical knowledge and practical application. Laboratory staff will undergo biosafety-related training at least once a month, ensuring a continuous improvement in their understanding of the latest biosafety standards and practices. Additionally, quarterly simulation exercises will be conducted to refine the laboratory’s emergency response plans, and improvements will be documented as formal procedural guidelines to standardize and formalize the emergency response processes. In special circumstances, such as the emergence of a new infectious disease or system upgrades, the frequency of training and exercises will be increased accordingly to ensure that staff are equipped with the latest response strategies and techniques. These regular trainings and drills not only keep the staff’s skills up-to-date and reinforced but also help them respond quickly and effectively in the event of a public health emergency. Through a series of simulation exercises, staff will become familiar with system operations and will continuously adjust and optimize workflows in practice, ultimately achieving optimal prevention and control outcomes. Moreover, the system’s design incorporates continuous improvement needs, with regular system evaluations and feedback to continually adjust and optimize system settings and operational protocols, ensuring long-term effectiveness and adaptability. This ongoing evaluation and improvement mechanism is a highlight of the system’s design, ensuring it remains up-to-date and adaptable to the evolving needs and challenges of medical practice (Figure 5).

### 4.4 Professional capability enhancement and knowledge innovation

#### 4.4.1 Off-site advanced studies and professional training

To elevate the overall level of laboratory biosafety management, biosafety administrators are sent to superior hospitals for further education. Through extensive training and observation, they acquire cutting-edge theoretical knowledge and management skills, focusing particularly on the following areas: ① Biosafety Policy Development: Administrators gain an in-depth understanding of how superior hospitals formulate biosafety policies, which cover personnel protection, sample handling, pathogen testing, and isolation. They also learn how to apply international standards and best practices across different laboratory levels. ② Risk Assessment Processes: Administrators thoroughly learn advanced risk assessment processes, including dynamic monitoring, data analysis, weight allocation, and report generation. They master the art of appropriately allocating weights to different risk levels and adjust standard operating procedures (SOPs) based on actual laboratory operations. ③ Emergency Response and Training Systems: Observing the entire process of emergency drills, administrators learn how to simulate various types of sudden incidents to ensure that emergency plans can handle all emergencies. Additionally, they understand how training systems maintain laboratory staff’s efficiency in emergency responses through regular drills and theoretical instruction. ④ Biosafety Facility Design and Maintenance: By examining the design and maintenance processes of biosafety facilities in different laboratories, administrators learn how to select appropriate ventilation systems, sterilization equipment, and bio-protection equipment, along with methods for their regular inspection and maintenance.

#### 4.4.2 Strategy review and update

Upon returning, administrators integrate the newly acquired knowledge and experience with existing strategies to optimize the laboratory biosafety management system, with significant updates in the following areas: ① Risk Assessment Process: Inspired by advanced experiences from superior hospitals, the risk assessment process is rewritten to prioritize emerging pathogens and high-risk operations. A dynamic monitoring mechanism is introduced to adjust the weights and standards of different risk indicators through continuous data analysis, making the process more timely. ② Emergency Response and Training Drills: A more detailed emergency response strategy is formulated, ensuring that every employee understands their responsibilities in various emergencies. The training and drill programs are optimized to closely integrate regular drills with actual workflow, simulating real infectious disease outbreaks to enable rapid and correct responses by all staff. ③ Biosafety Policy and Facility Management: Biosafety policies are readjusted according to international standards to ensure that operations in each department comply with the new strategy. A regular inspection and maintenance regime is introduced to ensure the effective operation of every biosafety facility.

### 4.5 Construction and execution of the departmental biosafety assessment system

To ensure that laboratory biosafety measures are fully implemented and continuously improved based on evaluation results, the design of the assessment system must consider comprehensiveness, objectivity, and operability (28).

#### 4.5.1 Design and process establishment of the assessment manual

The assessment manual encompasses critical elements of biosafety, including risk assessment, equipment management, operational standards, emergency response, and training effectiveness. The evaluation process is divided into three stages to ensure a comprehensive and objective assessment. Initially, the self-assessment phase allows two infection control administrators in the laboratory to thoroughly inspect the implementation of each biosafety element and accurately record related data. Subsequently, members of the quality management team perform a cross-check of the biosafety conditions within the laboratory to ensure the neutrality and accuracy of the assessment. Finally, professionals from the infection control department conduct a final review to validate the results of the self-assessment and cross-check, providing professional feedback to ensure the coherence and accuracy of the evaluation results.

#### 4.5.2 Assessment effectiveness and continuous improvement strategies

**Figure 6:**
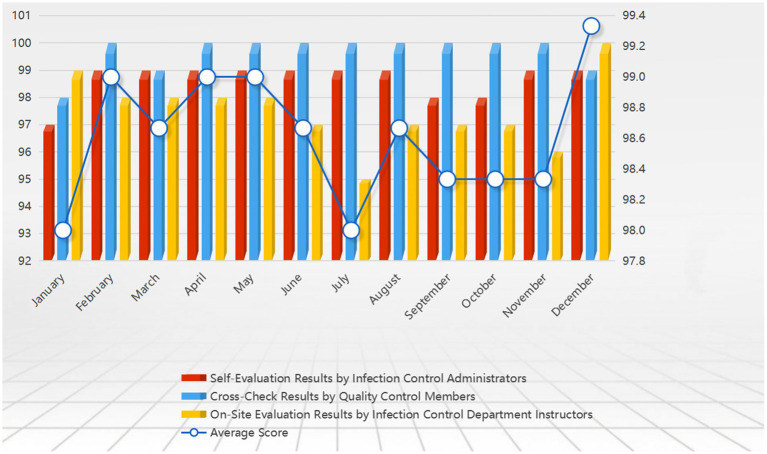
Shows the 2023 laboratory biosafety evaluation results, with scores out of 100. It compares system functions and emergency response indicators before and after the implementation of the major respiratory infectious disease hospital infection control system. The figure highlights improvements in real-time data monitoring, automated risk assessments, and emergency resource management, demonstrating the enhanced efficiency and effectiveness of the new system.

During the assessment process, data from each phase is promptly collected, and dynamic feedback mechanisms are used to continuously optimize the design and procedures of the assessment manual. Continuous improvement strategies are developed based on the evaluation results, linking identified issues with the laboratory’s Standard Operating Procedures (SOPs) for biosafety. Through regular monthly reviews, the biosafety compliance and operational execution of each laboratory are aligned with internal benchmarks and international standards. In the final review process, professionals from the infection control department provide feedback to laboratory leaders, emphasizing the importance of improvement strategies and offering practical guidance. Regular reviews and feedback ensure the consistency of quality oversight and the ongoing implementation of improvement measures, further enhancing the overall level of laboratory biosafety (Figure 6).

## 5 Conclusion

This study has accomplished a paradigm shift in laboratory biosafety management, evolving from a mere biosafety manual to a comprehensive quality management system. The quality management system encompasses four levels: strategy, procedures, operations, and records. It ensures that laboratory operational processes are standardized and consistent from overarching strategies to Standard Operating Procedures (SOPs), thereby enhancing the execution of biosafety management while strengthening the staff’s awareness of standards and operational capabilities.

In terms of the comprehensive update of the risk assessment system, the new system adopts a multi-dimensional comprehensive assessment approach that includes microbial attributes, operational complexity, laboratory environment, and personnel qualifications. This approach facilitates a thorough identification of potential biological threats. The new assessment system dynamically adjusts assessment standards to continuously improve the identification capabilities for high-risk pathogens and provides laboratory managers with reliable risk level guidance. The combined use of comprehensive assessment methods and dynamic adjustment mechanisms significantly enhances the laboratory’s capabilities in early warning and prevention of biological threats.

In response to significant respiratory diseases, this study introduced the “Psychiatric Medical Institution Major Respiratory Disease Infection Control System, “which encompasses simulation drills, real-time feedback, and effectiveness tracking modules to comprehensively enhance staff biosafety awareness and emergency response capabilities. The system integrates data-driven decision support and real-time operational optimization, significantly enhancing the laboratory’s response capabilities in the face of emergencies. Due to its advanced design and practical effectiveness, the project consecutively won the top two positions in the “Maker China Entrepreneurship Tianfu” competition held by the Sichuan Provincial Department of Science and Technology for 3 years and won the first prize in the 2021 Chengdu Medical Science and Technology Award (Project number: 2020-YF05-00171-SN). Moving forward, we will continue to research and optimize the system in biosafety management and emergency response, setting higher infection control standards for psychiatric medical institutions. This innovative system will equip psychiatric medical institutions with enhanced biosafety management and emergency response capabilities, ensuring rapid, scientific, and effective decisions in the face of complex public health events.

Through professional development and knowledge innovation, external training and professional education have enabled laboratory personnel to master the latest biosafety theories and operational skills, significantly boosting their confidence and proficiency in emergency handling. By integrating the latest theories with practical experience, the laboratory’s biosafety management has seen continuous improvement and enhancement.

The structured design and scoring system of the self-assessment manual ensure ongoing improvements in the laboratory, while the cross-departmental collaboration mechanism enables real-time resource sharing and risk monitoring, further enhancing the overall level of biosafety management in the laboratory.

**Figure 7:**
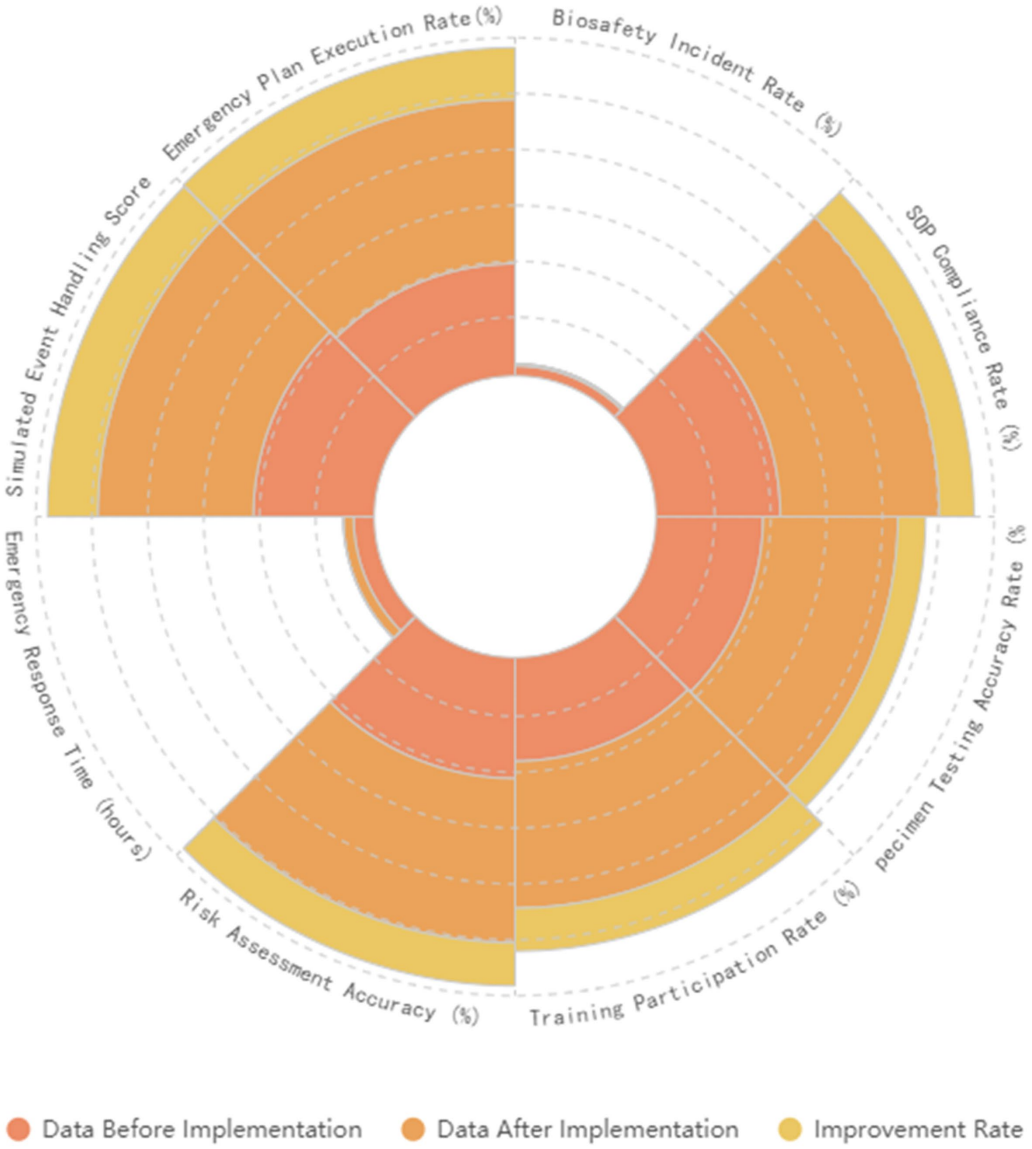
Shows the comparison of results before and after the implementation of the comprehensive strategy. It compares various biosafety assessments and emergency response indicators before and after the implementation.

In the future, we will continue to refine these improvement measures by monitoring and evaluating the effectiveness of our systems, integrating the latest scientific research and technological developments to further enhance our biosafety management systems. Additionally, we plan to explore the use of artificial intelligence and big data technologies to improve the efficiency and accuracy of risk assessment and management. We will also strengthen training and practical operation drills to continuously elevate the level of biosafety management in our laboratories (Figure 7).

## 6 Discussion

In this current study, we have implemented a series of improvements that significantly enhanced the effectiveness of laboratory biosafety management. However, given the rapid development of biotechnology and the unpredictability of global pandemics, it is crucial to further deepen and expand our research directions.

Firstly, although we have expanded the scope of risk assessment and successfully enhanced the identification of potential biological hazards, the existing assessment methods still face challenges in adapting to rapidly changing environments. To improve the predictive and responsive capabilities toward biosafety threats, we plan to integrate real-time data and artificial intelligence algorithms into the risk assessment framework, aiming for more efficient threat monitoring through dynamic and predictive analysis. This system is expected to combine machine learning models, epidemiological data, and laboratory operation records to construct an intelligent system capable of proactively detecting potential threats, predicting risk levels, and triggering early warnings (26). However, in practice, the quality and efficiency of data collection and processing still face technical and resource constraints. High-quality data collection relies on precise laboratory records and data input, where any minor negligence can significantly impact the accuracy of assessment results. Furthermore, although artificial intelligence algorithms offer new possibilities for predicting unknown risks, the construction and training of these algorithms depend on extensive data and complex adjustment processes, which are particularly challenging in resource-limited laboratory environments. These limitations suggest that we need to explore more effective data management and algorithm optimization methods in future research to ensure the practicality and reliability of the risk assessment system (27).

Secondly, the emergency drill system introduced has significantly improved our emergency response capabilities, but the effectiveness of the system highly depends on the realism of the drill design and the participation of the employees. To further enhance this effect, we suggest the adoption of Virtual Reality (VR) and Augmented Reality (AR) technologies to create more realistic emergency scenarios and enhance the immersive learning experience for employees. These high-tech methods simulate various actual biosafety incidents, allowing employees to gain valuable hands-on experience in a virtual environment, thus enhancing their rapid response capabilities in real operations (28). However, despite the more realistic environments provided by VR and AR technologies, their high cost and maintenance requirements limit their feasibility for broader application. Moreover, although virtual drills can provide complex simulation environments, they cannot completely replicate all possible scenarios and emergency conditions in the real world, which May affect the comprehensiveness and practicality of the drills. Therefore, future challenges include balancing the application of technology with cost-effectiveness and ensuring that virtual drills comprehensively cover all potential biosafety threats to ensure the practicality and continuous optimization of drill content (29).

Third, the self-assessment manual, although it provides a structure for continuous improvement, its effectiveness May be limited by subjective evaluations. We recommend introducing 360-degree feedback and Key Performance Indicators (KPIs) assessment methods to comprehensively measure the overall performance of employees in biosafety management and combine these with quantitative performance indicators to achieve more precise self-assessment and improvement (30). However, the effectiveness of this method largely depends on the honest feedback and active participation of employees. If employees lack sufficient identification with the process or feel pressured by the assessment process, it May affect the authenticity and completeness of the feedback (33).

Finally, cross-departmental collaboration is crucial for biosafety management, yet establishing an effective cooperation mechanism remains a challenge. Adopting a network governance model will optimize the collaboration structure, ensuring the flow and sharing of resources and information, and further enhancing the efficiency of interdepartmental cooperation. This approach allows for a seamless management system where information flows smoothly between departments, forming a rapid-response joint action team (31). However, practical implementation May encounter issues such as interdepartmental conflicts of interest, insufficient collaboration, or information silos. Ensuring effective communication and resource sharing between departments requires continuous organizational commitment and cultural adjustment (34).

In summary, this study has made significant progress in enhancing laboratory biosafety, but by introducing dynamic assessments, high-tech drills, objective self-assessments, and networked collaboration, the laboratory biosafety management system can be further optimized. This prepares for future biosafety challenges with a more solid, flexible, and efficient foundation. Simultaneously, it is necessary to integrate relevant theoretical frameworks, promote interdisciplinary cooperation, continuously explore new biosafety management strategies, and apply intelligent technologies to all aspects of biosafety to address the increasingly complex and variable global biosafety landscape.
